# Soluble Aβ aggregates can inhibit prion propagation

**DOI:** 10.1098/rsob.170158

**Published:** 2017-11-15

**Authors:** Claire J. Sarell, Emma Quarterman, Daniel C.-M. Yip, Cassandra Terry, Andrew J. Nicoll, Jonathan D. F. Wadsworth, Mark A. Farrow, Dominic M. Walsh, John Collinge

**Affiliations:** 1 MRC Prion Unit at UCL, UCL Institute of Prion Diseases, National Hospital for Neurology and Neurosurgery, Queen Square, London WC1N 3BG, UK; 2 Laboratory for Neurodegenerative Research, Ann Romney Center for Neurologic Diseases, Brigham and Women's Hospital, Harvard Medical School, Boston, MA 02115, USA

**Keywords:** amyloid β-protein, Alzheimer's disease, automated scrapie cell assay, Creutzfeldt–Jakob disease, prion

## Abstract

Mammalian prions cause lethal neurodegenerative diseases such as Creutzfeldt–Jakob disease (CJD) and consist of multi-chain assemblies of misfolded cellular prion protein (PrP^C^). Ligands that bind to PrP^C^ can inhibit prion propagation and neurotoxicity. Extensive prior work established that certain soluble assemblies of the Alzheimer's disease (AD)-associated amyloid β-protein (Aβ) can tightly bind to PrP^C^, and that this interaction may be relevant to their toxicity in AD. Here, we investigated whether such soluble Aβ assemblies might, conversely, have an inhibitory effect on prion propagation. Using cellular models of prion infection and propagation and distinct Aβ preparations, we found that the form of Aβ assemblies which most avidly bound to PrP *in vitro* also inhibited prion infection and propagation. By contrast, forms of Aβ which exhibit little or no binding to PrP were unable to attenuate prion propagation. These data suggest that soluble aggregates of Aβ can compete with prions for binding to PrP^C^ and emphasize the bidirectional nature of the interplay between Aβ and PrP^C^ in Alzheimer's and prion diseases. Such inhibitory effects of Aβ on prion propagation may contribute to the apparent fall-off in the incidence of sporadic CJD at advanced age where cerebral Aβ deposition is common.

## 1. Introduction

Prion diseases are fatal neurodegenerative disorders associated with propagation of multi-chain assemblies of misfolded cellular prion protein (PrP^C^) [1,2]. Prions propagate by recruitment of α-helical-rich PrP^C^ into β-sheet-rich infectious rod-like structures [3,4]. In addition to serving as the precursor of infectious prions, expression of PrP^C^ is also required for the neurotoxicity in prion infection [5–8].

Numerous studies suggest PrP^C^ may play a role in Alzheimer's disease (AD), and there is evidence that PrP can modulate the production, aggregation and toxicity of the amyloid β-protein (Aβ) [9–11]. In 2009, Lauren *et al*. [11] reported that a preparation of aggregated synthetic Aβ1-42 known as Aβ-derived diffusible ligands (ADDLs), which contained a mixture of globular oligomers and protofibrils, bound to PrP^C^. Using a series of deletion constructs and anti-PrP antibodies, it was shown that PrP residues 95–110 were required for ADDL binding. In accord with this finding, the authors demonstrated that knock-out of the mouse PrP gene (*Prnp*) or pre-treatment of hippocampal slices with an antibody directed to PrP_93-109_ protected against ADDL-induced synaptotoxicity. These provocative findings were followed by multiple *in vivo* and *in vitro* studies, most of which supported a role for PrP^C^ in aspects of Aβ-mediated toxicity [10,12–21]. However, others have reported deleterious effects of Aβ that do not require PrP^C^ expression [22–25]. Recently, we reported that only certain Aβ assemblies exert toxicity in a PrP-dependent fashion which may explain some of these apparently discrepant findings [26].

However, all published studies that have examined binding of Aβ to PrP^C^ agree that there is high affinity and specific binding for soluble aggregates [11,13,21–23,26–29], and high-resolution analysis suggests that binding of Aβ occurs at two sites: centred around residues approximately 23–33 and approximately 88–113 [29]. Although initially surprising, the finding that PrP^C^ can serve as an acceptor for soluble aggregates of Aβ [11] is consistent with the hypothesis that the unstructured N-terminus (encompassing residues approx. 23–128) of PrP^C^ acts as a molecular sensor which can interact with a broad range of ligands [30], including other β-sheet-rich oligomeric proteins [28]. Moreover, the same binding sites for soluble aggregates of Aβ have previously been shown to also be important for binding of prions to PrP^C^ [31–36].

While many studies have investigated the interaction between Aβ and PrP^C^, and how it might contribute to AD pathogenesis, there has been little research on whether Aβ binding to PrP^C^ can affect prion propagation. Here, we used the well-established cell-based prion bioassay (the scrapie cell assay using PK1/2 neuroblastoma-derived cells) [37] and a chronically prion-infected cell line (iPK1/2 cells) [38,39] to address these critical issues. We found that soluble Aβ aggregates (ADDLs), but not Aβ monomers or fibrils, could prevent infection of PK1/2 cells when ADDLs were co-administered with the prion inoculum. Strikingly, when added to iPK1/2 cells already chronically infected with prions, ADDLs had a marked cell curing effect. This protective effect appears to be mediated by ADDL binding to PrP^C^. While diverse studies have linked PrP to AD [9,11,40–43], our data raise the possibility that soluble Aβ aggregates may actually protect against prion disease. Thus, whether Aβ binding to PrP^C^ has pathogenic or protective effects may depend on the relative concentrations of relevant Aβ and PrP assemblies.

## 2. Material and methods

### 2.1. Reagents

All chemicals and reagents were purchased from Sigma-Aldrich unless otherwise noted. Synthetic Aβ1-42 and Aβ1-40 were synthesized and purified using reversed-phase HPLC by Dr James I. Elliott at the ERI Amyloid Laboratory (Oxford, CT, USA). Peptide mass and purity (greater than 99%) were confirmed by reversed-phase HPLC and electrospray/ion trap mass spectrometry. All tissue culture reagents were obtained from Invitrogen.

### 2.2. Aβ preparations

Aβ is prone to aggregate and can form an array of different assemblies. In this study, we used conditions to yield preparations highly enriched in: (i) monomers, (ii) pre-fibrillar aggregates, known as ADDLs and (iii) amyloid fibrils. Monomeric Aβ was prepared by dissolving dry Aβ1–40 peptide at 2 mg ml^−1^ in 6 M guanidine hydrochloride (GuHCl) and then subjecting this preparation to asymmetric flow field-flow fractionation (AFFFF). The AFFFF channel was eluted in 50 mM ammonium bicarbonate pH 8.5, and fractions containing monomeric Aβ, as judged by molar mass (approx. 4000 g mol^−1^), were collected and immediately frozen at −80°C. ADDLs were prepared essentially as described previously [19], approximately 25 mg of Aβ1–42 peptide was dissolved in anhydrous DMSO, gently rocked for approximately 5 min and then diluted to 0.5 mg ml^−1^ in phenol red-free Ham's F12 medium without l-glutamine (Caisson Labs) and incubated quiescently at room temperature (RT). At approximately 6 h intervals, aliquots were removed, briefly centrifuged at 16 100*g* and analysed using AFFFF. Typically, at 24–36 h, less than 20% of the injected mass eluted as monomer, as judged by the area under the curve of both monomer and oligomer peaks. Thereafter, the material was aliquoted and stored frozen at −80°C. To form fibrils, Aβ was solubilized and incubated as for ADDLs, but the incubation continued for 30 days. For cell culture experiments, Aβ preparations were buffer exchanged into Opti-MEM using a centrifugal concentrator (Amicon, Ultra 0.5 ml, 5 K cut-off).

### 2.3. Asymmetric flow field-flow fractionation and multi-angle light scattering

Experiments were conducted using a 24.6 cm long channel fitted with a 350 µm spacer and a 5 kDa MWCO polyethersulfone membrane. Aliquots of Aβ preparations (190 µl) were injected onto an Eclipse DualTec AFFFF (Wyatt Technology, Santa Barbara, CA, USA) and eluted with 50 mM ammonium acetate pH 8.5. The sample was injected at 0.2 ml min^−1^, followed by a 1 min focusing period, and then eluted with a 1.5 ml min^−1^ cross-flow for 45 min. Light scattering was performed using a Wyatt Dawn Heleos II multi-angle light scattering module to calculate the molar mass.

### 2.4. Electron microscopy

Negative stain electron microscopy (EM) was performed as described previously [26]. Peptide solutions (5 µl) were loaded onto negatively charged glow-discharged copper grids coated with a continuous carbon film. Samples were left to adhere for 120 s and excess solution blotted with grade 4 Whatman paper. Thereafter, grids were stained with 2% uranyl acetate for 40 s, blotted and air-dried. Images were acquired on an FEI Tecnai T10 electron microscope operating at 100 kV and recorded on a 1 k × 1 k charge-coupled device camera (Gatan) at a typical magnification of 34 000 with a pixel size of 5 Å.

Prion rods were purified as described previously [4,44] concentrated to 100× (relative to starting 10% brain homogenate) and mixed with 10 µM ADDLs and incubated at 21°C for 1 h. Prion rods were pelleted by centrifugation at 16 100*g* and 25°C for 30 min. The pellet was washed once with Opti-MEM and centrifuged a final time. The pellet was resuspended in Opti-MEM to one half the volume of the starting prion/ADDL solution and stained for EM as described above. Images were analysed for evidence of ADDLs binding to prion rods. First, the number of protofibrillar and spherical Aβ species in an area containing a rod cluster were counted, then the number of Aβ species in an equivalent sized area that did not include prion rods were counted. This was repeated for three rod clusters and three rod-free areas, and images were analysed by two different users.

### 2.5. Automated scrapie cell assay

An automated version of the standard scrapie cell assay (SCA) using PK1/2 cells [45] was used as described previously [37,46]. Briefly, PK1/2 cells were grown in Opti-MEM, containing 10% fetal calf serum; 100 U ml^−1^ penicillin and 100 µg ml^−1^ streptomycin at 37°C, 5% CO_2_. Twenty-four hours before infection with Rocky Mountain Laboratory (RML), PK1/2 cells were seeded in 96-well plates at 18 000 cells per well and grown in Opti-MEM. ADDLs or bovine serum albumin (BSA) were incubated with RML prion-infected brain homogenate (I-BH) (designated I8700; [44]) for 1 h at RT and then added to cells and incubated for 72 h. Thereafter, cells were split 1 : 8 into fresh cell culture media containing fetal calf serum and grown to confluence. Two further passages were conducted, removing initial inoculum, before transferring a sample of the cells to enzyme-linked immunospot (ELISPOT) plates for measurement of the number of prion-infected cells (identified by detection of proteinase K-resistant PrP, PrP^Sc^), the ‘spot count’ [45]. The viability of the cells was monitored using Trypan Blue. Prion titre in the experimental samples was determined by reference to a calibration curve in each experiment derived from a serial dilution of an RML brain homogenate of known prion titre (10^8.3^ intracerebral LD_50_ units g^−1^ brain) determined by prior mouse bioassay [37,44].

The ability of ADDLs to retard prion propagation was calculated relative to the number of infected cells ‘spot count’ of cells incubated with the equivalent RML concentration alone (positive control) and the ‘spot count’ (background noise) of cells incubated without RML present.

### 2.6. Curing assay of chronically Rocky Mountain Laboratory prion-infected cells

Chronically RML prion-infected PK1/2 (designated iPK1/2) cells were used to assay curing activity. As described previously, these cells are able to maintain a robust prion infection long term in culture [38,39,47]. Briefly, iPK1/2 cells were produced by incubating cells with 1 × 10^−3^ RML-I-BH for 72 h [44]. Thereafter, cells were passaged every 2–3 days for 2 weeks to remove any remaining inoculum. A portion of infected cells was analysed for RML prion infectivity by ELISPOT and the remainder stored in liquid nitrogen. For experiments, cells were thawed and cultured as described above. In order to maintain a consistent level of prion infection, cells were never passaged more than 15 times.

iPK1/2 cells were seeded at 6000 cells per well, in 384-well plates. The cells were grown in Opti-MEM for 3 days at 37°C and 5% CO_2_ ± Aβ. Additionally, positive (2 µM 5000 Da dextran sulfate) and negative (cells only) controls were included on each plate. Infected cells produce PrP^Sc^. On day 4, cells were analysed for both viability and PrP^Sc^ content. Cell viability was assessed using the CellTiter-Glo Luminescent assay (Promega) and PrP^Sc^ levels were measured by dot blot. For PrP^Sc^ analysis, the media was removed from the cells, lysis buffer added (Tris buffer pH: 7.5 with NaCl, Triton X-100 and sodium deoxycholate) and the cells lysed on ice for 20 min. The lysates were mixed and bound to nitrocellulose membrane, using a 96-well bio-dot microfiltration apparatus (BioRad). The samples were treated with proteinase K (5 µg ml^−1^) for 1 h at 37°C and then denatured using 3 M guanidine thiocyanate. PrP^Sc^ was detected using the anti-PrP antibody ICSM18 (D-Gen Ltd, London) and goat anti-mouse IgG-IRDye 800CW (LI-COR Biosciences, Santa Clara, CA, USA). Spots were visualized using an Odyssey infrared imaging system (LI-COR Biosciences) and the relative intensity of the infrared signal was determined using the systems software.

### 2.7. Immunofluorescence

PK1/2 cells were seeded at 18 000 cells per coverslip in 24-well plates. The cells were grown in serum-free media for 3 days at 37°C and 5% CO_2_ ± Aβ. On day 4, coverslips were washed three times with PBS and then fixed in 4% PFA at RT for 15 min. After fixation, coverslips were washed twice with PBS and then blocked with 5% BSA/PBS (1 h at RT) and stained. Cells were incubated for 1 h at RT with the anti-PrP antibody ICSM18 (1.25 µg ml^−1^) and/or the anti-Aβ rabbit antiserum #2454 at 1 : 2000 dilution (Cell Signalling, Danvers, MA, USA). Thereafter, cells were washed with PBS and then incubated with Alexa Fluor 488 tagged donkey anti-mouse IgG (H + L) (#A-21 202) at 2 µg ml^−1^ and/or Alexa Fluor 546-tagged donkey anti-rabbit IgG (H + L) (#A10040) at 3.3 µg ml^−1^ (Invitrogen Life Technologies). Nuclei were stained with 4,6-diamidino-2-phenylindole, 1 µg ml^−1^ for 1 h at RT. Cells were washed with PBS and then mounted using fluorescence mounting medium (DAKO). Images were captured using a Zeiss LSM710 confocal laser scanning microscope and co-localization quantified using Volocity 3D imaging software (Perkin Elmer).

## 3. Results

### 3.1. Aβ-derived diffusible ligands inhibit prion propagation and cure prion infection

ADDLs are a polydisperse solution of soluble Aβ aggregates which include globular oligomers, protofibrils and monomer [11,48] and bind to the PrP^C^ specifically and with high affinity [11,13,21,26,27,29]. Two regions of PrP^C^ (one centred around residues 23–33 and the other around 88–113) are particularly important for Aβ binding [11,13,27,29], and these are the same sites thought to be important for PrP^Sc^ binding to PrP^c^ [31–36]. Thus, we sought to determine if ADDLs could compete with prions for binding to PrP^C^ and attenuate prion propagation.

**Figure 1.**
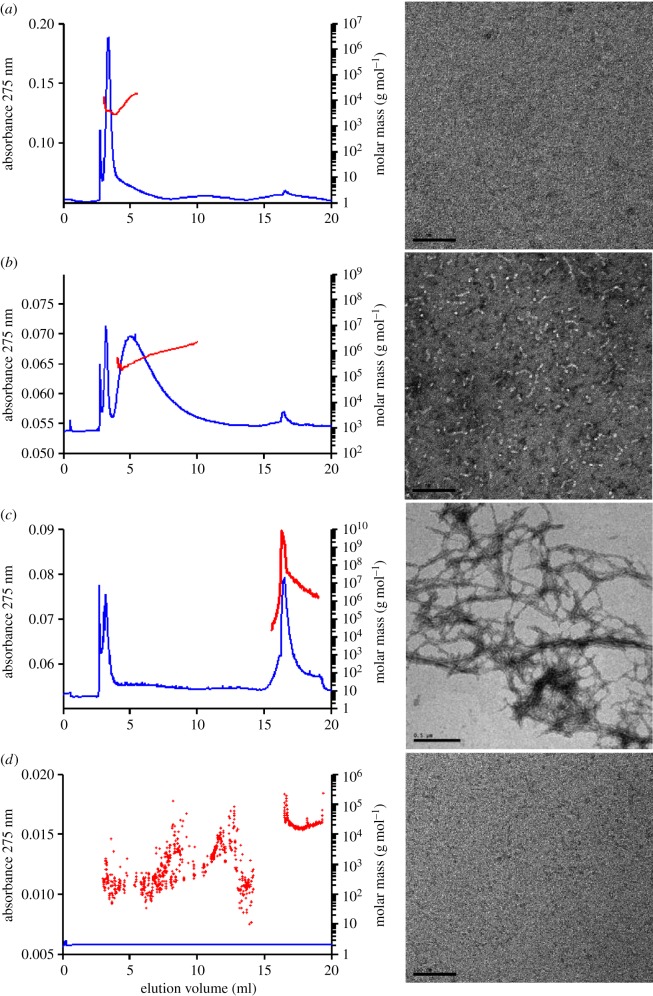
Characterization of Aβ species. AFFFF is a chromatography technique in which the separation of the sample is performed in a channel rather than a column, and separation of differently sized particles is achieved with two perpendicular flow directions of elution buffer. In AFFFF, small particles elute first, thus the elution profile is the inverse order of size exclusion chromatography. About 190 µl of each Aβ preparation was injected and eluted in 50 mM ammonium acetate pH 8.5 at 1 ml min^−1^. Each AFFFF plot shows absorbance at 275 nm throughout the run (blue line) and molar mass (red dots) across the main UV peak. Only the main UV peak contained enough protein for the molar mass to be accurately calculated—see the molar mass of the buffer-only sample (*d*) as an example. Data are shown for Aβ40 monomer (*a*), ADDLs (*b*), Aβ42 fibrils (*c*) and Opti-MEM only (*d*). The EM image for each sample is also shown. Scale bars: (*a*,*b*,*d*) 100 nm, (*c*) 500 nm.

As ADDLs are known to bind with high affinity to PrP^C^, whereas Aβ monomers show little or no binding and fibrils exhibit only weak binding [26], we generated ADDLs from Aβ42 and relatively homogeneous preparations of Aβ40 monomers and Aβ42 fibrils, and characterized each using AFFFF and EM (figure 1). AFFFF is a flow-based method in which separation takes place in a channel where sample retention is caused by the action of a cross-flow that is generated by a second independent stream that runs across the channel at right angles to the primary channel flow [49]. Unlike more commonly used size exclusion chromatography, in AFFFF, small particles elute earlier than larger particles. AFFFF of ADDLs confirmed the presence of a small amount of monomer and a range of Aβ assemblies with molar masses from 300 000 to 3 000 000 g mol^−1^ (figure 1*b*). EM also indicated that ADDLs contained a mixture of structures, including imperfect spheres of approximately 5–10 nm diameter and abundant protofibrils (flexible fibrils) of approximately 5–10 nm diameter and less than 100 nm in length. By contrast, our monomer and fibril preparations were relatively homogeneous. Monomer preparations had a molar mass of 4000–5000 g mol^−1^ (figure 1*a*) and contained no structures detectable by EM, whereas fibrils had a molar mass of greater than 10^9^ g mol^−1^ and formed complex latticeworks of long fibrils with diameters of approximately 10 nm (figure 1*c*).

**Figure 2.**
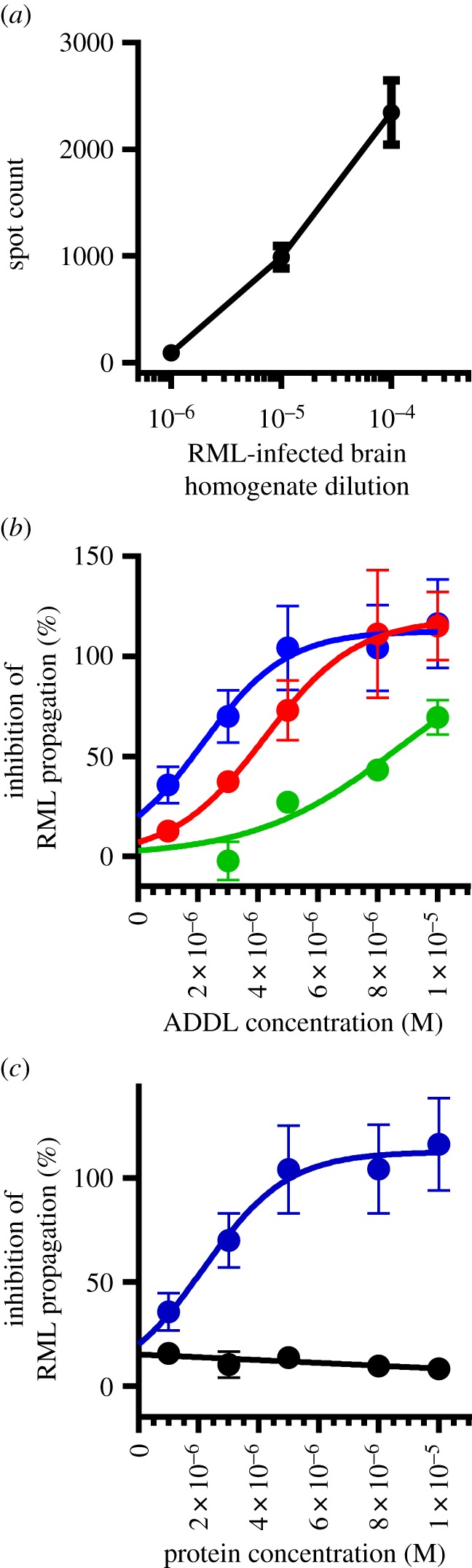
ADDLs inhibit prion propagation in the scrapie cell assay (SCA). ADDLs were incubated with RML prions for 1 h before addition to PK1/2 cells. Every 2–3 days, the cells were split 1 : 8 and passaging was repeated three times. After each passage, the viability and amount of infection of the cells was assessed by trypan blue and ELISPOT revelation, respectively. (*a*) The spot count of prion-infected PK1/2 cells increases with increasing concentration of prion containing brain homogenate. (*b*) PK1/2 cells incubated with a serial dilution of ADDLs and either 3 × 10^−6^ (blue curve), 1 × 10^−5^ (red curve) or 1 × 10^−4^ (green curve) diluted RML-I-BH. (*c*) About 3 × 10^−6^ RML homogenate incubated with a serial dilution of either ADDLs (blue curve) or BSA (black curve). ADDL concentration is based on the monomer equivalent concentration. Data shown are the mean and standard deviation of six replicates.

To test if ADDLs could attenuate prion infectivity, we used an automated high-throughput prion bioassay referred to as the automated crapie cell assay (ASCA) [37,45,50,51]. PK1/2 cells were incubated with an RML prion-I-BH [44]. Cells were grown to confluence, split 1 : 8 and grown to confluency again. The cycle of growth and passage was repeated a further two times to remove initial infecting inoculum, and confluent cells from the third passage were used to measure the proportion of infected cells [37]. As expected, the extent of prion infection is strongly influenced by the dilution of I-BH, with lower dilutions resulting in more infected cells over the course of the SCA (figure 2*a*). To determine if ADDLs could attenuate prion infection, three dilutions of I-BH (3 × 10^−6^, 1 × 10^−5^, 1 × 10^−4^) were incubated with a range of ADDLs concentrations (1–10 µM) for 1 h and then added to cells (figure 2*b*). The dilutions of I-BH were chosen to yield optimal spot counts within the linear dynamic range of the ELISPOT reader (see Material and methods) [37]. Importantly, addition of ADDLs to the inoculum caused a dose-dependent decrease in the extent of prion propagation, an effect that was directly related to the prion titre in the starting inoculum (figure 2*b*). For instance, when I-BH was used at a dilution of 3 × 10^−6^ (blue curve), maximal inhibition of infectivity was achieved with a dose of 5 µM ADDLs, whereas when more concentrated I-BH (1 × 10^−4^, green curve) was used, higher concentrations of ADDLs were required to significantly attenuate prion propagation (figure 2*b*). In comparison, addition of BSA had no effect on prion propagation (black curve, figure 2*c*).

**Figure 3.**
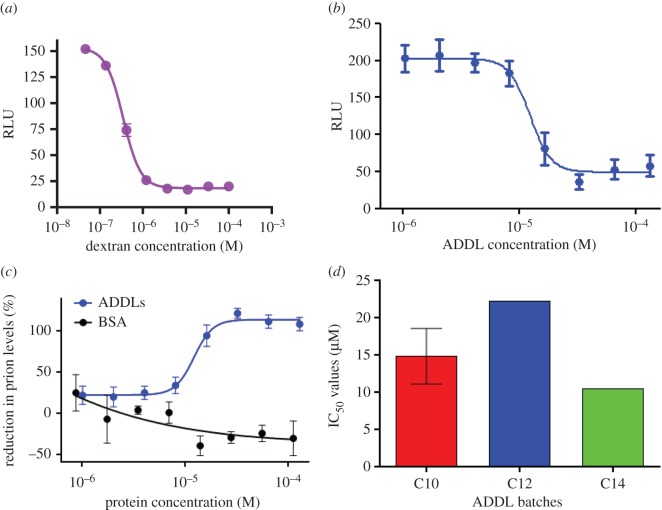
Treatment of chronically prion-infected cells with Aβ. (*a*) iPK1/2 cells are a subline of neuroblastoma N2a cells, which maintain a stable infection of RML prions. Infected cells produce PrP^Sc^ without cytotoxicity. The cells were incubated with or without ADDLs for 72 h, then overall levels of PrP^Sc^ are detected by immunoblot. (*a*,*b*) Raw luminescence units (RLUs) demonstrating the decrease in infected cells with increasing concentrations of (*a*) dextran sulfate or (*b*) ADDLs. (*c*) Inhibition of PrP^Sc^ propagation by ADDLs (blue) is not seen with the same concentration of BSA (black) at concentrations between 1 and 100 µM. Both datasets are expressed as percentage reduction in PrP^Sc^ levels relative to the reduction by 2 × 10^−6^ M dextran sulfate. A reduction in PrP^Sc^ levels in this assay correlates with reduction in prion levels assessed by bioassay. Mean and standard deviation shown, *n* = 3. (*d*) Comparison of IC_50_ values for three different preparations of ADDLs in the chronically prion-infected cell assay. The mean and s.d. of *n* = 4 for C10 is shown (*n* = 1 for C12 and C14).

Next, we investigated if ADDLs could cure cells with an established chronic prion infection. Chronically infected PK1/2 cells (iPK1/2 cells) accumulate PrP^Sc^ yet remain viable (see Material and methods) and have been successfully used in drug screening to identify anti-prion compounds [38,39,47]. Dextran sulfate is effective at curing prion infection in this assay and has been used a positive control in drug screening [39,52] and was used as a comparator to assess the inhibitory activity of ADDLs across experiments. iPK1/2 cells were incubated with increasing concentrations of dextran sulfate for 72 h (the maximum time before the cells require passage) and the levels of PrP^Sc^ were measured by dot blot. As expected, dextran sulfate caused a dose-dependent decrease in PrP^Sc^ with an apparent IC_50_ of approximately 5 × 10^−7^ M and apparent curing at concentrations greater than 1 × 10^−6^ M (figure 3*a*). Incubation of iPK1/2 cells under the same conditions, but this time in the presence of increasing concentrations of ADDLs also resulted in a dose-dependent reduction of PrP^Sc^ with apparent IC_50_ approximately 2 × 10^−5^ M and apparent curing at concentrations greater than 5 × 10^−5^ M (figure 3*b*). To allow comparison between experiments, we expressed the curing ability of ADDLs relative to the levels of PrP^Sc^-treated cells plus and minus 2 × 10^−6^ M dextran sulfate (figure 3*c*). As in the ASCA assay (figure 2), ADDLs caused a dose-dependent decrease in the levels of PrP^Sc^, whereas BSA did not (figure 3*c*). The ability of ADDLs to cure chronic prion infection was consistent across experiments when the same preparation of ADDLs was used (figure 3*d*, preparation C10) and when two other ADDL preparations were tested (figure 3*d*, preparations C12 and C14). The IC_50_ for ADDLs used in six different experiments ranged from 10.4 to 22.2 µM (figure 3*d*).

### 3.2. Aβ-derived diffusible ligands, but not Aβ monomers or fibrils inhibit prion propagation

**Figure 4.**
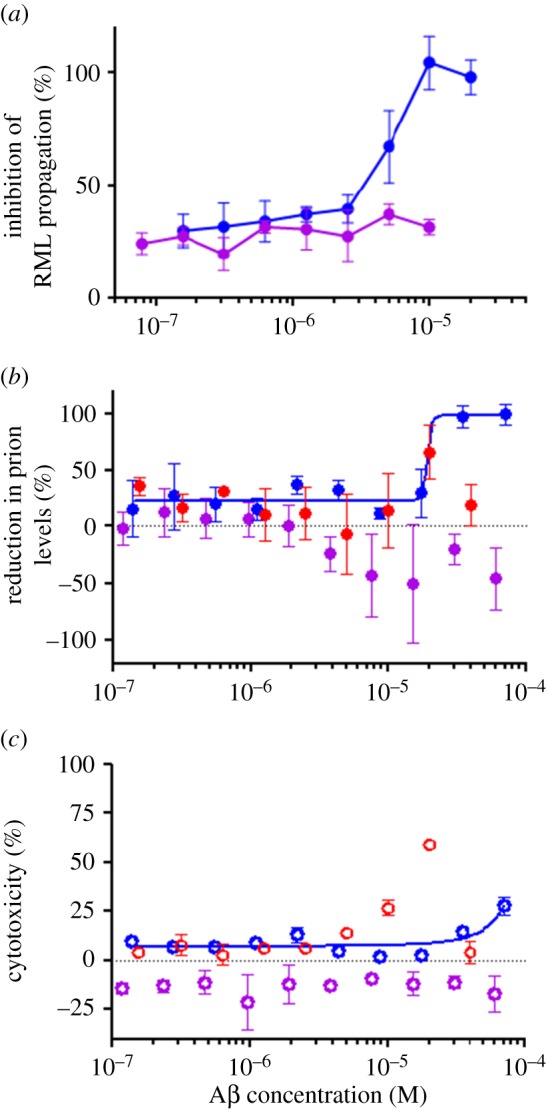
ADDLs, Aβ monomer and fibrils were tested for their ability to cure prion infection. (*a*) ASCA data for the effect of Aβ40 monomer (purple) and ADDLs (blue) on RML propagation. Cells were treated with a 3 × 10^−6^ dilution of RML-I-BH. Mean and s.d. of *n* = 6 replicates shown. (*b*) A reduction in PrP^Sc^ levels (corresponding to reduction in prion titre) by Aβ40 monomer (purple), Aβ42 fibrils (red) and ADDLs (blue) using the chronically prion-infected cell assay (*n* = 3). (*c*) As in (*b*) except measuring cytotoxicity determined by the CellTiter-Glo Cell Viability assay. Mean of *n* = 2 replicates shown.

Previous studies found that Aβ monomers do not bind to PrP^C^ and that Aβ fibrils bind to PrP^C^ less well than pre-fibrillar intermediates [11,26,27], hence we sought to determine if there was a relationship between the ability of different Aβ structures to bind to PrP^C^ and their ability to reduce prion propagation (figure 4). First, we examined if Aβ40 monomer could influence prion propagation in the ASCA. As expected, ADDLs caused a dose-dependent decrease in prion propagation, whereas Aβ monomers had no effect (figure 4*a*). To determine if this prion-curing activity was similarly specific for pre-fibrillar Aβ species, we compared the effects of ADDLs versus Aβ40 monomers and Aβ42 fibrils using the chronic prion-infected cell assay. As before (figure 4*b,c*), ADDLs caused a dose-dependent decrease in prion infection (blue solid circles), whereas Aβ monomers and fibrils had no effect (figure 4*b*). Given that reduction in detectable PrP^Sc^ could occur due to cell loss, and that Aβ is known to be toxic to certain cells, we were careful to measure cell viability in all of the cultures treated with Aβ. The number of metabolically active cells (as assessed by the CellTiter-Glo Luminescent Cell Viability assay) did not change over the concentration range at which ADDLs inhibited prion infectivity (less than or equal to 2 × 10^−5^ M; figure 4*c*). Therefore, the reduction in PrP^Sc^ levels mediated by ADDLs is not a consequence of cell compromise, but rather a specific effect comparable to that seen with other prion-curing agents [39,52,53].

### 3.3. Aβ-derived diffusible ligands bind to the surface of PrP^C^-expressing cells, but not to purified prion rods

**Figure 5.**
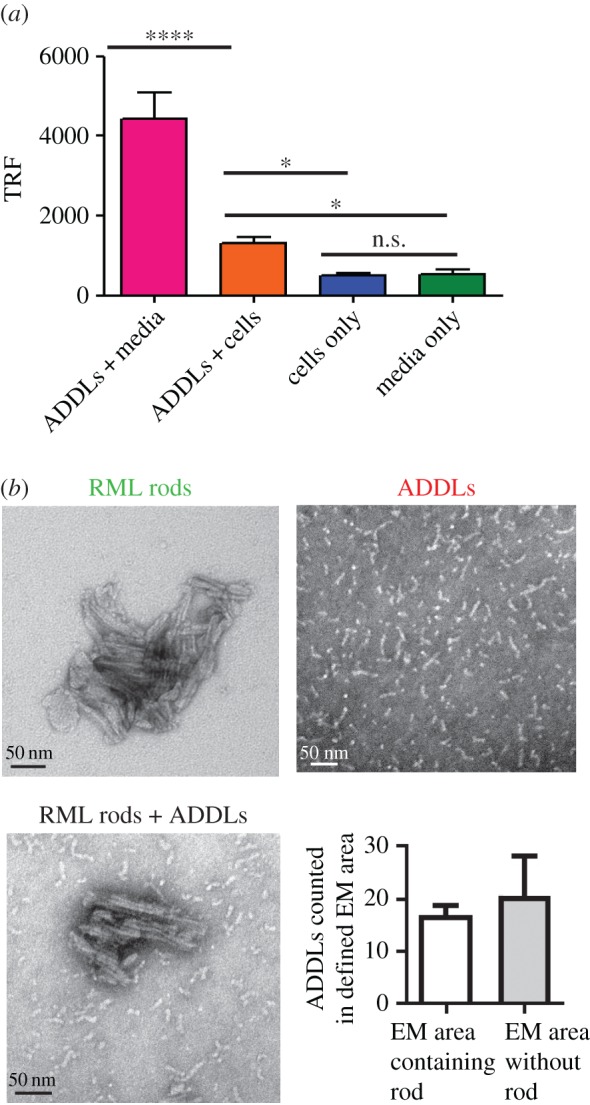
ADDLs associate with PrP^c^ on the cell surface but do not appear to interact with PrP^Sc^. (*a*) Fewer ADDLs (*p* < 0.0001) are detectable in cell media by PrP-82E1 DELFIA after 72 h when incubated with cells compared to incubation in cell media only. Mean and s.d. of *n* = 4 replicates shown, univariate ANOVA. (*b*) Infectious RML prion rods were purified from RML-infected mouse brain [4,44] and incubated with 10 µM ADDLs for 1 h before imaging by EM. The number of Aβ oligomers was counted in equivalent areas containing rods or without rods in three EM images of different RML rod-Aβ oligomer clusters by two different users. No enrichment of Aβ oligomers on the rods surface was observed (non-significant, *p* = 0.456, unpaired *t*-test). Scale bars, 50 nm. *p ≤ 0.05; ****p ≤ 0.0001.

As both PrP^C^ and PrP^Sc^ share the same primary structure, including the amino acids that comprise the Aβ binding sites [27,29], we investigated whether the prion inhibition we observed was due to Aβ acting on PrP^C^ or prions, or both. If ADDLs inhibited infectivity by binding to prions, then it should be possible to detect ADDLs bound to PrP^Sc^. To address this issue, we incubated highly purified infectious prion rods [4,44] with ADDLs under the same conditions used in the ASCA, and then searched for binding of ADDLs to prions using negative stain EM (figure 5). Both prion rods and ADDLs were readily detected, but we saw no evidence of co-localization. This rather rudimentary assay provides the first evidence that Aβ does not bind to PrP^Sc^.

**Figure 6.**
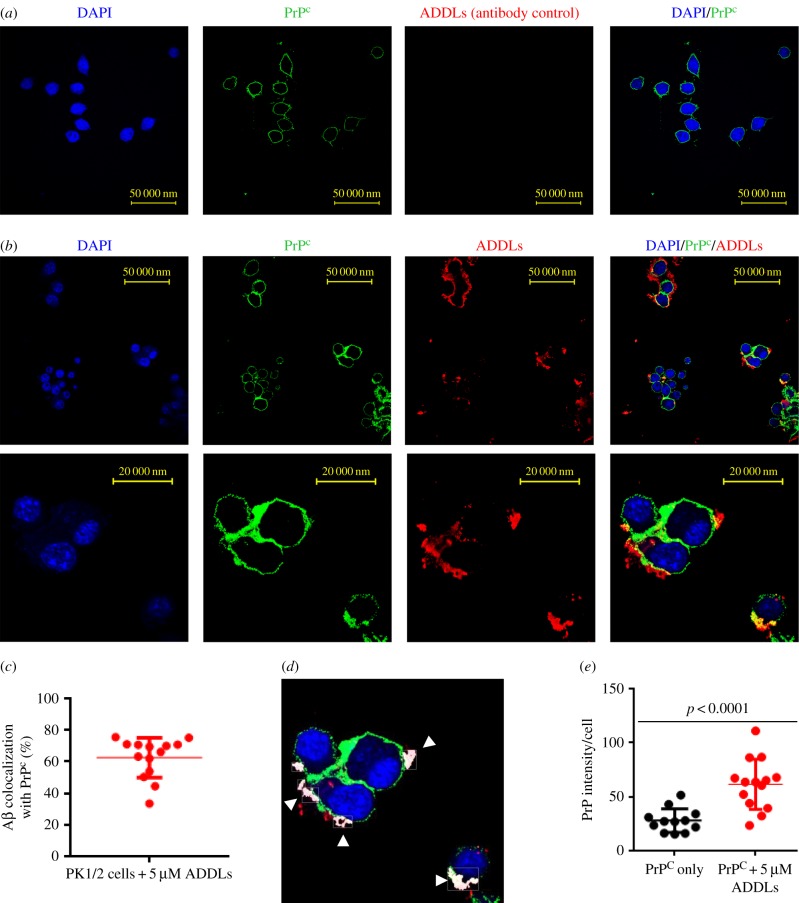
ADDLs and PrP^c^ co-localize at the cell membrane and PrP^c^ levels are increased. (*a*) PK1/2 cells incubated without ADDLs were immunostained with DAPI (blue) or anti-PrP antibody ICSM18 (green) or anti-Aβ antibody 2454 (red) and imaged by confocal microscopy (40× magnification). The anti-Aβ antibody 2454 was included as an antibody control. (*b*) Immunostaining of the cells after 3 days incubation with 5 µM ADDLs show co-localization of PrP^c^ and ADDLs at the cell membrane. (*c*) About 63 ± 3% of ADDLs co-localize with PrP^c^ on the cell surface. (*d*) Volocity analysis image; areas of PrP/Aβ co-localization (highlighted in white) indicated by arrows. (*e*) Quantification of PrP^c^ on the cell surface of PK1/2 cells shows a significant (*p* < 0.0001) increase in PrP^c^ intensity on the surface of non-permeabilized cells incubated with 5 µM ADDLs (62 ± 6, *n* = 14) than without ADDLs (28 ± 3, *n* = 12) (mean and s.d., two-tailed Student's *t*-test).

As ADDLs did not seem to interact with prion rods, we looked for evidence of ADDL binding to PrP^C^ on the surface of PK1/2 cells. Immunostaining of non-permeabilized cells detected Aβ on the surface of PK1/2 cells and partial co-localization with PrP^C^ (figure 6*a–c*). Interestingly, we also observed that treating PK1/2 cells with ADDLs increased cell surface levels of PrP^C^ (figure 6*b,d*). In accord with earlier reports, ADDLs appear capable of binding to PrP^C^ [11,15] and retaining PrP^C^ at the plasma membrane [54]. Thus, it seems likely that ADDLs inhibit prion levels and propagation by competing with prions for binding to PrP^C^, and may also retard internalization of PrP^C^.

## 4. Discussion

Persuasive evidence from multiple investigators argues that certain soluble assemblies of Aβ can bind tightly to PrP^C^ [11,13,21–23,26–29]. The interaction between PrP and soluble Aβ aggregates is highly specific [11,13,26,27,29] and involves sites previously implicated in binding of PrP^Sc^ [31–36]. Attention has focused on how this interaction may contribute to AD pathogenesis, but Aβ binding to PrP^C^ also has implications for prion diseases. PrP^C^ is the obligate substrate for prion propagation and is essential for neurotoxicity [2,6,8,55] and agents that bind to PrP^C^ have the potential to modulate infectivity and toxicity [39,56].

Here, we show that ADDLs inhibit prion infectivity in a dose-dependent manner and reduced the levels of proteinase K-resistant PrP in chronically prion-infected cells. As both PrP^C^ and PrP^Sc^ have the same primary structure, including the sites involved in ADDL binding, the ability of ADDLs to attenuate prion propagation could result from interactions involving either PrP^C^ or PrP^Sc^. When ADDLs were mixed with highly purified prions, we found no evidence of binding, whereas when ADDLs were added to PrP^C^-expressing cells ADDLs partially co-localized with cell surface PrP^C^. Moreover, as we and others have shown previously [11,13,21,26,27], and we confirmed for preparations used in this study (data not shown), recombinant monomeric PrP (rPrP) readily binds ADDLs. Taken together, these data suggest that ADDLs can attenuate prion infectivity by directly binding to PrP^C^ and acting as a competitive inhibitor (electronic supplementary material, figure S1). Such a mechanism would allow the cells' natural prion clearance rate [57] to outpace any residual propagation, resulting in the low to absent levels of PrP^Sc^ observed when ADDLs were used in our experiments. Consistent with this mechanism, we also found that Aβ species (monomer and fibrils) which show little or no affinity for monomeric PrP lack the ability to attenuate prion propagation.

The binding response between rPrP and ADDLs indicates an apparent dissociation constant of approximately 100 nM, whereas the IC_50_ of ADDLs in the chronic prion-infected cell assay was approximately 15 µM. The difference between binding to rPrP and the ability to inhibit prion propagation probably results because (i) ADDLs are known to bind non-PrP membrane components [11], (ii) our assays use mitotic cells which have a doubling time of approximately 24 h and (iii) ADDLs are competing with prions for binding to PrP^C^. In terms of ADDLs, the concentration that might be needed to attenuate prion formation *in vivo*, it is worth considering that the amount of ADDLs used in our experiments are expressed as monomer equivalents, yet we know that the component of ADDLs that binds to PrP has a relatively high molecular weight and only contributes a fraction of the total Aβ present [26]. Therefore, the actual *K*_D_ for the binding component of ADDLs must be significantly lower, and may be in the picomolar range. As to how much ADDLs would be required to inhibit prion propagation *in vivo*, that will depend on the amount of infectious prions.

Our findings are in apparent conflict with a prior study that reported prion inoculation of Tg2576 APP transgenic mice accelerated both Aβ deposition and prion disease [58]. A possible explanation for the divergence in results seen with Tg2576 mice and those we detected in PK1 cells relates to the forms of Aβ tested in our study and those produced by Tg2576 mice. We and others have previously shown that only certain forms of Aβ bind to PrP [11,13] and that only particular effects of Aβ are mediated by PrP [26]. Similarly, it is known that certain APP transgenic mice exhibit cognitive phenotypes that depend on the expression of PrP, whereas others do not [16,25]. In terms of the acceleration of prion disease in Tg2576 mice, it is interesting to note that deleting PrP^C^ expression in Tg2576 results in only a partial rescue of cognitive performance as opposed to the complete recovery seen in other APP transgenic lines [59]. Further, Tg2576 mice have been shown to produce little or no Aβ species capable of binding to PrP [59]. Given that Tg2576 mice show minimal PrP-dependent deficits and produce little Aβ that binds PrP, it is perhaps not surprising that Tg2576 mice are unable to attenuate prion infectivity and propagation.

Clearly, high concentrations of ADDLs should completely inhibit prion propagation, but they are also expected to cause neuronal dysfunction. Thus, high levels of soluble Aβ assemblies may provide relative protection from human prion disease, but cause AD. The lack of co-localization of disease-associated PrP and Aβ deposits seen in a recent study is of interest in this regard [60]. These observations support the notion that soluble aggregates of Aβ and PrP may compete for binding to PrP^C^ *in vivo* and that the balance between the levels of these aggregates is a critical determinant of whether and what form of neurodegenerative disease will result. The most common human prion disease, sporadic Creutzfeldt–Jakob disease, which has a relatively uniform incidence worldwide and apparently random population distribution, is thought to represent the spontaneous production of prions as a rare stochastic event [61,62]. In this regard, it has always been intriguing why its apparent incidence falls at advanced age (greater than 80 years) [63,64]. While this may in part be due to lower diagnosis rates in the elderly, it is conceivable that this could also be related to the common occurrence of Aβ deposition in this age group.

## Supplementary Material

Putative Mechanism for ADDL inhibition of RML propagation.

## Data accessibility

All relevant data are presented in figures in the manuscript and electronic supplementary material.
