# Supplementary material for: Soluble Aβ aggregates can inhibit prion propagation
Source: Open Biol. 2017 Nov 15;7(11):170158. doi: 10.1098/rsob.170158 (PMC5717343; doi:10.1098/rsob.170158)
Supplement: Putative Mechanism for ADDL inhibition of RML propagation. [file rsob170158supp1.pdf]

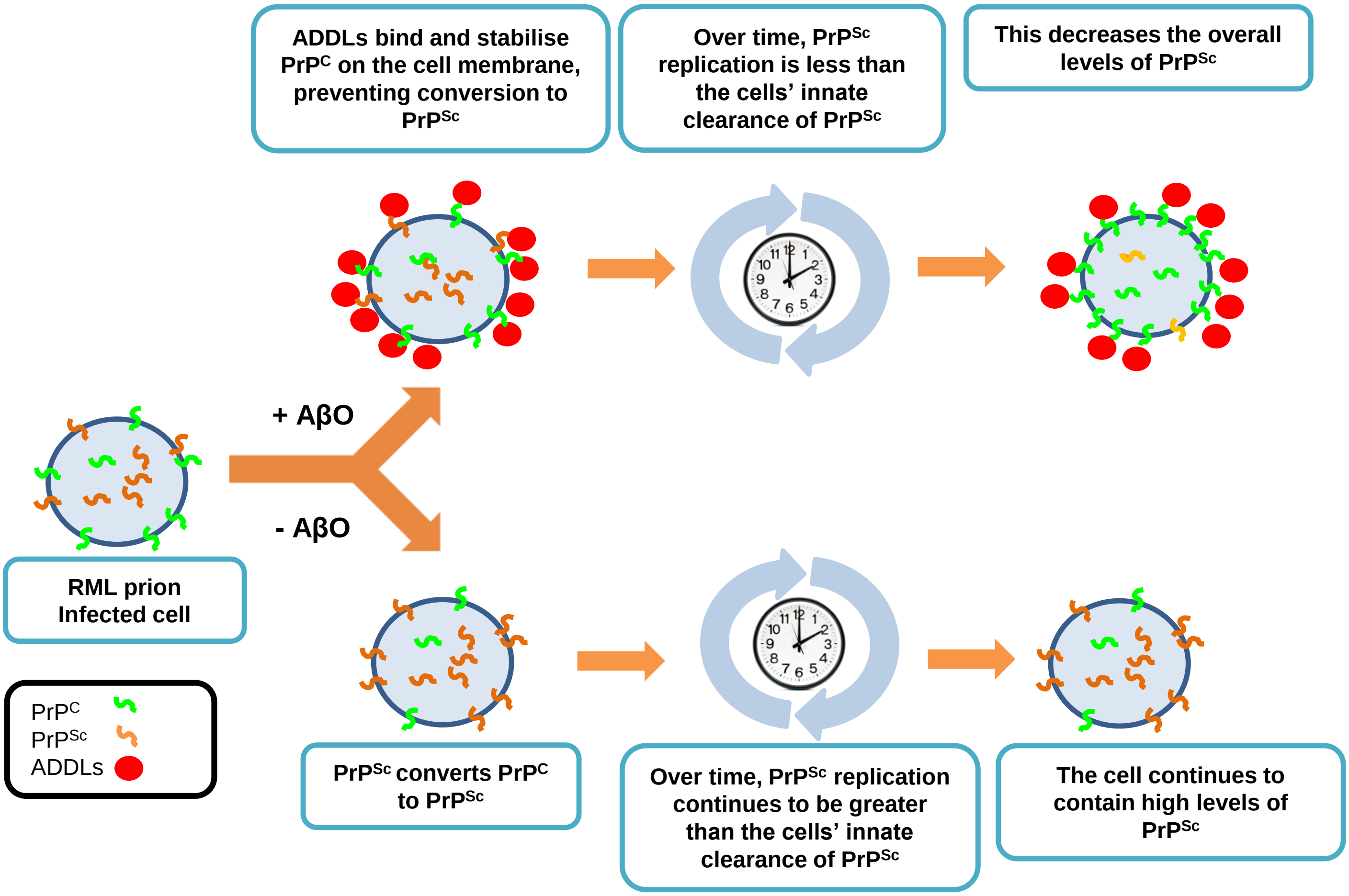
ADDLs bind and stabilise PrP^C on the cell membrane, preventing conversion to PrP^{Sc}
Over time, PrP^{Sc} replication is less than the cells' innate clearance of PrP^{Sc}
This decreases the overall levels of PrP^{Sc}
+ AβO
- AβO
RML prion Infected cell
PrP^C
PrP^{Sc}
ADDLs
PrP^{Sc} converts PrP^C to PrP^{Sc}
Over time, PrP^{Sc} replication continues to be greater than the cells' innate clearance of PrP^{Sc}
The cell continues to contain high levels of PrP^{Sc}
